# Modeling the Full Time-Dependent Phenomenology of Filled Rubber for Use in Anti-Vibration Design

**DOI:** 10.3390/polym12040841

**Published:** 2020-04-06

**Authors:** Francesca Carleo, Jan Plagge, Roly Whear, James Busfield, Manfred Klüppel

**Affiliations:** 1 School of Engineering and Materials Science, Queen Mary University of London, Mile End Road, London E1 4NS, UK; f.carleo@qmul.ac.uk; 2 Deutsches Institut für Kautschuktechnologie e.V., Eupener Str. 33, 30519 Hannover, Germany; jan.plagge@dikautschuk.de (J.P.); manfred.klueppel@dikautschuk.de (M.K.); 3 Jaguar Land Rover, Banbury Road, Gaydon CV35 0RR, UK; rwhear1@jaguarlandrover.com

**Keywords:** viscoelastic behaviour, modelling, elastomer, cyclic softening

## Abstract

Component design of rubber-based anti-vibration devices remains a challenge, since there is a lack of predictive models in the typical regimes encountered by anti-vibration devices that are deformed to medium dynamic strains (0.5 to 3.5) at medium strain rates (0.5/s to 10/s). An approach is proposed that demonstrates all non-linear viscoelastic effects such as hysteresis and cyclic stress softening. As it is based on a free-energy, it is fast and easily implementable. The fitting parameters behave meaningfully when changing the filler volume fraction. The model was implemented for use in the commercial finite element software ABAQUS. Examples of how to fit experimental data and simulations for a variety of carbon black filled natural rubber compounds are presented.

## 1. Introduction

Suspension is one of the most important systems affecting a vehicle’s ride quality, and it is, therefore, a key factor in determining a vehicle’s performance. The modelling of bushing force in the automotive industry plays an important role in predicting the dynamic behaviour of the suspension system. The viscous behaviour of elastomers makes the use of rubber components essential to reduce the level of vibrations that are transmitted to the passengers in the vehicle. In particular, natural rubber is the material of choice for these types of engineering applications. For automotive suspension components, carbon black is also typically used as a filler to improve the rubber’s mechanical properties such as the fatigue behaviour or the stiffness. For bushing applications, quasi-static deformations up to large strains are of major interest. In these particular conditions, the rubber exhibits non-linear viscoelastic behaviour such as the Mullins Effect, cyclic stress softening, hysteresis and induced anisotropy [1,2,3,4,5]. This characteristic response is related to the molecular microstructure, but it is not yet totally understood [6]. Furthermore, these effects are also present in unfilled strain-crystallizing elastomers as natural rubber [7,8,9,10]. Modelling of filled rubber introduces difficulties related to its nonlinear and incompressible behaviour. Many models try to capture this behavior from a range of different perspectives. The multiscale nature of polymers is reflected by different computational methods for specific length and time scales: quantum (∼$10^{-10}$ m, ∼$10^{-12}$ s), atomistic (∼$10^{-9}$ m, ∼$10^{-9}$–$10^{-6}$ s), mesoscopic (∼$10^{-6}$ m, ∼$10^{-6}$–$10^{-3}$ s) or macroscopic scale (∼$10^{-3}$ m, ∼1 s) [11,12,13]. At the top level, phenomenological macroscopic models can roughly be classified into three categories [14] damage models, rheological models with serial and parallel combination of elastic and viscous elements and the constitutive equations based on a rubber elasticity model. Damage models [15,16,17] are unable to differentiate between an unloading and a subsequent reloading with one consequence being that they cannot predict the cyclic stress softening phenomenon. Essentially, there is a limitation in fitting the experimental behaviour as a limitation of the background theory. Rheological Framework models are models with elastic and viscous components. The Parallel Rheological Framework ([18,19]) is capable of reproducing the full range of nonlinear viscoelastic effects that are under examination, but it requires a large number of parameters that are very sensitive to the input test data. The dynamic flocculation model (DFM) assumes the breakdown and reformation of filler aggregates as the main mechanism [20]. Other physically-based works focus on the binding or sliding of polymer chains on the fillers surface [21]. Recent Molecular Dynamics (MD) simulations indicate that both phenomena may play a role [22,23].

Most of these models include softening effects using non-time dependent mathematics, e.g., by softening the material based on maximum strain or stress measures. In contrast, experiments clearly show that softening proceeds by repeatedly stretching the material to a predefined load (cyclic stress softening) or by holding it at constant stretch (stress relaxation) [24]. More specifically, stress decreases logarithmically [25,26,27]. In a recent review article [14] it was found that no predictive model was able to fully reproduce cyclic stress softening to a satisfying quality. In the next sections a new approach based on a recently proposed model [28] is presented. It is able to account for non-linear elasticity and strain history effects.

## 2. Theory

### 2.1. Free Energy Density

The underlying free energy density is derived on the basis of the extended non-affine tube model of rubber elasticity [29,30] which was shown to be the best compromise between fitting quality and number of parameters [31]. In a simplified form it reads

(1) $$W\left({\tilde{I}}_{1},{\tilde{I}}^{*}\right)=\frac{G_{c}}{2}\frac{{\tilde{I}}_{1}}{1-\frac{1}{n}{\tilde{I}}_{1}}+2G_{e}\;{\tilde{I}}^{*}$$

with

(2) $$\begin{array}{cc} {\tilde{I}}_{1} & =I_{1}-3=\lambda_{1}^{2}+\lambda_{2}^{2}+\lambda_{3}^{2}-3 \end{array}$$

(3) $$\begin{array}{cc} {\tilde{I}}^{*} & =I^{*}-3=\lambda_{1}^{-1}+\lambda_{2}^{-1}+\lambda_{3}^{-1}-3 \end{array}$$

being modified invariants of the left Cauchy Green tensor, expressed by the principal stretches $\lambda_{i}$. The straightforward simplifications done to arrive at Equation (1) are outlined in [32]. The parameter $G_{c}$ is called crosslink modulus and scales the contribution of crosslinks to the mechanical response. Accordingly, $G_{e}$ scales proportionally to the number of trapped entanglements of the polymer [33]. The third parameter *n* measures the number of statistical segments between network nodes, which is a measure of elastically effective chain length. For example, natural rubber has a segment length of about 0.934 nm [34], corresponding to roughly two isoprene units. Fillers are introduced by assuming that they amplify strain heterogeneously within the matrix by local amplification factors *X* [28]. To avoid problems arising from frame references the amplification factors directly act on the invariants. In the case of ${\tilde{I}}_{1}$ this is reasonable, as it represents the norm of a hypothetical length within the material. The amplified energy density is then calculated as a superposition of differently amplified domains

(4) $$W_{X}\left({\tilde{I}}_{1},X_{\max},X_{\min}\right)=\int_{X_{\min}}^{X_{\max}}dX\;P\left(X\right)\;W\left(X{\tilde{I}}_{1},X{\tilde{I}}^{*}\right)$$

where the distribution of amplification factors is given by

(5) $$P\left(X\right)={\left(X+C\right)}^{-\chi }\;\cdot \;\frac{\chi -1}{{\left(X_{\min}+C\right)}^{1-\chi }-{\left(X_{\max}+C\right)}^{1-\chi }}$$

The parameter χ gives the width of the distribution and *C* defines a plateau value around X=1. For C=0 Equation (5) is equivalent to its equivalent in [28]. It is normalized to the interval [$X_{\min}$,$X_{\max}$], which is motivated from conservation of the number of rubber-filler structures. Carrying out the integral given by Equation (4) generates hypergeometrical functions. Additive splitting of the integrand, as is explained in [32], gives a good approximation containing only elementary functions:

(6) $$W_{X}\left(X_{\max},X_{\min}\right)\approx \frac{1}{2}\frac{1}{{\left(X_{\min}+C\right)}^{1-\chi }-{\left(X_{\max}+C\right)}^{1-\chi }}\;\cdot \;\left[\frac{G_{c}\;{\tilde{I}}_{1}+4\;G_{e}\;{\tilde{I}}^{*}}{\chi -2}\left({\left(C+X_{\min}\right)}^{1-\chi }\left(C+\left(\chi -1\right)\;X_{\min}\right)-{\left(C+X_{\max}\right)}^{1-\chi }\left(C+\left(\chi -1\right)\;X_{\max}\right)\right)\right.\left.+G_{c}\;n^{2}\left(\chi -1\right)\;{\tilde{I}}_{1}^{\;\chi -1}{\left(C\;{\tilde{I}}_{1}+n\right)}^{-\chi }\log\left(\frac{{\tilde{I}}_{1}\;X_{\min}-n}{{\tilde{I}}_{1}\;X_{\max}-n}\right)\right]$$

From Equation (6) the hyperelastic stresses can be derived, if $X_{\min}$ and $X_{\max}$ are known. The minimum amplification factor is set to $X_{\min}=1$, which corresponds to the assumption that there are non-amplified domains (e.g., without filler) within the material.

### 2.2. Stress Softening

In [28] the maximum amplification factor is defined to be a monotonically decreasing function of the all-time maximum of the modified first invariant ${\tilde{I}}_{1}$. This naturally introduces immediate stress softening if the material surpasses the previous maximum strain. As described in the introduction stress softening is not immediate, but proceeds logarithmically or according to a slow powerlaw. Generally, logarithmic relaxation in stress *f*∼−logt can be generated by differential equations of the form df/dt∼−exp|f|. From a physical point of view this can be understood in terms of force-induced hopping over a potential barrier, as formulated by Kramers [35]. It is reasonable to assume that softening is predominantly happening in the most stretched domains. A crude approximation of the stress-scaling in this regime gives

(7) $$f∼\frac{1}{\langle \lambda \rangle }W\left(X{\tilde{I}}_{1},X{\tilde{I}}^{*}\right)\approx \frac{1}{\sqrt{{\tilde{I}}_{1}}}\frac{{\tilde{I}}_{1}\;X_{\max}}{1-\frac{1}{n}{\tilde{I}}_{1}\;X_{\max}}$$

where the entanglement part of Equation (1) was neglected, because its influence is small at high strains. The prefactor $\langle \lambda \rangle =\sqrt{{\tilde{I}}_{1}}$ is a frame independent measure of the systems stretch. Heuristically, stress is also directly scaled by the amplification factor, such that df/dt$∼dX_{\max}/dt$. Using again the logarithm-generating differential equation df/dt∼−exp|f| and introducing scaling constants $c_{1}$ and $c_{2}$ this gives

(8) $$\frac{dX_{\max}}{dt}=-\exp\left(-c_{2}+\frac{1}{c_{1}}\frac{\sqrt{{\tilde{I}}_{1}}\;X_{\max}}{1-\frac{1}{n}{\tilde{I}}_{1}\;X_{\max}}\right)\;\cdot \;\frac{1}{s}$$

The timescale of zero-load softening given as $\tau_{\mathrm{soft}}=\exp\left(c_{2}\right)\;s$ was put in the exponential for numerical reasons. In practice, it should be sufficiently long that no significant softening happens on the timescale of the simulation, if no load is imposed. This means that for ${\tilde{I}}_{1}\to 0$ the result of Equation (8) is usually small and the maximum amplification factor $X_{\max}$ stays almost constant. When the model approaches divergence at ${\tilde{I}}_{1}\;X_{\max}/n\to 1$ the second summand inside the exponential increases and induces a decrease of $X_{\max}$. It shall be noted that a more careful derivation was carried out in [32] which relates $c_{1}$ and $c_{2}$ to temperature and allows the modeling of Mullins effect recovery [6] at elevated temperatures.

### 2.3. Viscoelasticity

Hysteresis is modeled by a single Maxwell element. The problem treated in this work requires only a narrow range of strain rates such that this procedure is justified. Viscoelastic stress is evolved according to the differential equation

(9) $$\frac{d\sigma_{\mathrm{vis}}}{dt}=-\frac{1}{\tau_{c}}\sigma_{\mathrm{vis}}+\frac{d\sigma_{\mathrm{el}}}{dt}$$

where $\sigma_{\mathrm{vis}}$ is the viscoelastic stress contribution, $\tau_{c}$ is the relaxation time scale, and $\sigma_{\mathrm{el}}$ is the elastic Cauchy stress derived from the free energy density given by Equation (6).

### 2.4. Implementation

The model was implemented in Matlab for the hypothetical condition of fully incompressible material, considering one material point loaded cyclically in the uniaxial direction and in a compressible full three dimensional form for the finite element analysis. For incompressible materials $J=I_{3}=1$ and the Cauchy stress is related to the free energy density:

(10) $$\sigma_{\mathrm{el}}=-pI+2\left(\frac{\partial W_{X}}{\partial I_{1}}+\frac{\partial W_{X}}{\partial I^{*}}\right)B$$

The scalar *p* is the hydrostatic pressure (an indeterminate Lagrange multiplier) that can be determined only from the boundary conditions. B is the left Cauchy-Green deformation tensor. For compressible materials it is useful to split the deformation into distortional and volumetric (dilational) parts via a multiplicative decomposition.

(11) $$F=J^{1/3}\bar{F}$$

(12) $$B=J^{2/3}\bar{B}$$

(13) $$I_{1}=J^{2/3}{\bar{I}}_{1}$$

The $J^{1/3}$ and $J^{2/3}$ terms are related to the volume changes, while the $\bar{•}$ terms are a modified gradient and strain related to the distortional deformations. These decompositions express the stored energy as $W({\bar{I}}_{1}\left(I_{1},J\right),{\bar{I}}^{*}\left(I^{*},J\right),J)$, that can be decomposed into volumetric and isochoric (distortional) responses (Equation (14)) and give the Cauchy stress [36] as in Equation (15)

(14) $$W\left(B\right)=W_{\mathrm{vol}}\left(J\right)+W_{\mathrm{iso}}\left(\bar{B}\right).$$

(15) $$\sigma_{\mathrm{el}}\left({\bar{I}}_{1},{\bar{I}}^{*},J\right)=\frac{2}{J}\left[\frac{\partial W_{X}}{\partial {\bar{I}}_{1}}+\frac{\partial W_{X}}{\partial {\bar{I}}^{*}}\right]\bar{B}+k\left(J-1\right).$$

**Figure 1:**
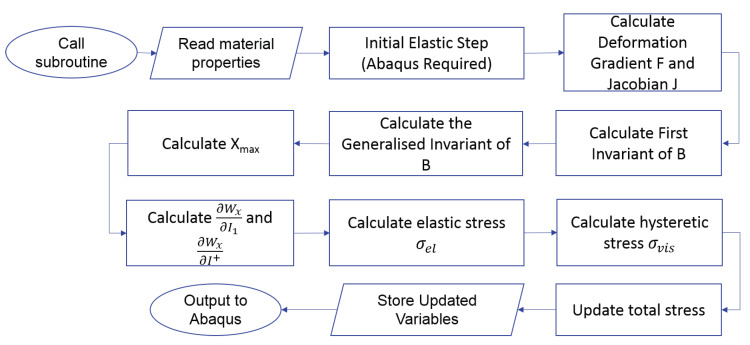
VUMAT Algorithm.

The single Prony series element was integrated explicitly and the total stress is defined as:

(16) $$\sigma_{\mathrm{tot}}=\left(1-\phi \right)\;\sigma_{\mathrm{el}}+\phi \;\sigma_{\mathrm{vis}}.$$

where ϕ is a fitting constant which is assumed to increase with filler volume fraction. Figure 1 shows the algorithm developed to implement the model as a user-defined material subroutine (VUMAT) coded in Fortran for Abaqus/Explicit.

The model requires 10 parameters, plus the bulk modulus in case of compressible material.

## 3. Materials and Experiments

Seven compounds supplied by TARRC (Tun Abdul Razak Research Centre, Brickendonbury, Hertford, UK), were examined. Each compound contained a different volume fraction of carbon black (FEF N550) that had been mixed into natural rubber (NR, SMR CV60). The compound formulations in phr (part per hundred of rubber by mass) are given in Table 1.

Characterisation of all this materials under cyclic tensile tests is available in [14] and it is briefly recalled in this section for the sake of completeness. Uniaxial tension tests were conducted using an Electropulse Instron Test Machine. Cyclic loading, unloading and reloading tests were performed using four different strain rates of 0.5/s, 1.5/s, 3/s and 6/s. All the specimens had a gauge length of 12 mm and a cross section of 2 mm × 0.5 mm. The nominal strain, $\varepsilon^{NOM}$, was determined as the ratio of the axial displacement to the original length of a specimen (12 mm). This test was difficult to conduct because the large strain amplitude and rate approached the limit of the test machine. To reach the desired strain rates, the specimens had to be short. In addition, the strain rate was too fast to be reliably measured using the optical strain measuring device. The tensile force was measured using a 1 kN load cell.

**Figure 2:**
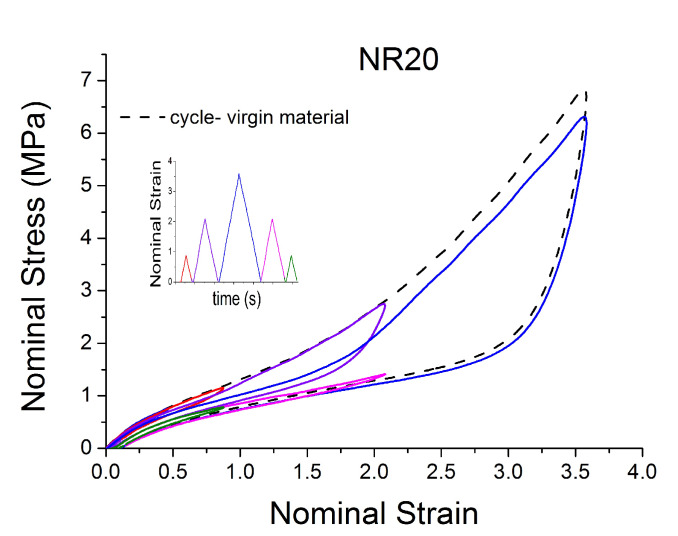
Stress–strain response of natural rubber filled with 20 phr of carbon black (NR20) when subjected to cyclic uniaxial tension. Different behaviour of the material after a different pre-strain is evident.

**Figure 3:**
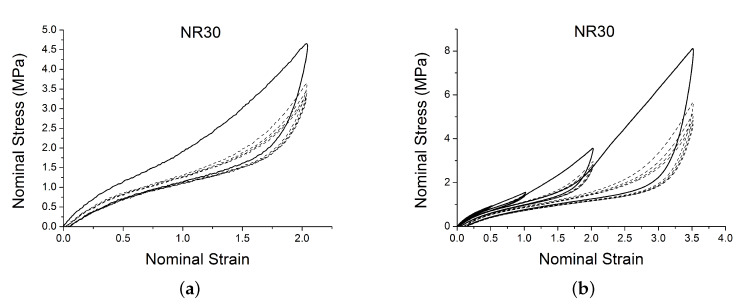
Stress–strain response of natural rubber filled with 30 phr of carbon black (NR30) submitted to cyclic uniaxial tension at 1.5/s. The solid curves are the trends for the first cycle at a given amplitude. The dotted curves are the trends after the initial cycle at given amplitude. (**a**) 5 cycles at the same amplitude, (**b**) 5 cycles at 4 different strain amplitude.

A subset of the results are shown in Figure 2 and Figure 3 for two of the materials. The results display strong nonlinearity, large hysteresis, complex Mullins behaviour (Figure 2 and Figure 3b), cyclic stress relaxation (Figure 3a) and permanent set. Full detail of all the experimental results can be found in [14].

In the range of interest the strain rate effect is not a dominant feature, so in the following analysis it was ignored.

## 4. Results

### 4.1. Sensitivity Analysis

This section shows how each parameter effects the output stress. The initial parameters shown in Table 2 are used, it is an arbitrary set. The parameter $X_{\max,0}=X_{\max}\left(t=0\right)$ is the boundary condition of the differential Equation (8).

**Figure 4:**
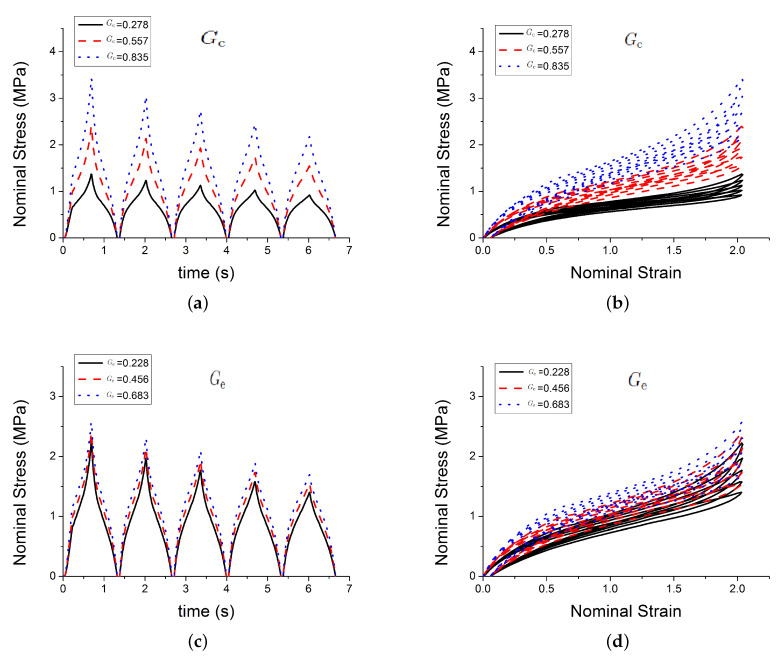

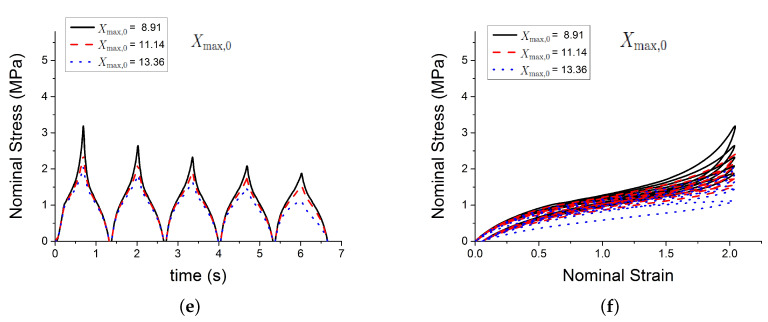
Influence of parameters. (**a**,**b**) show the effect of the parameter $G_{c}$; (**c**,**d**) show the effect of the parameter $G_{e}$; (**e**,**f**) show the effect of the parameter $X_{\max,0}$.

**Figure 5:**
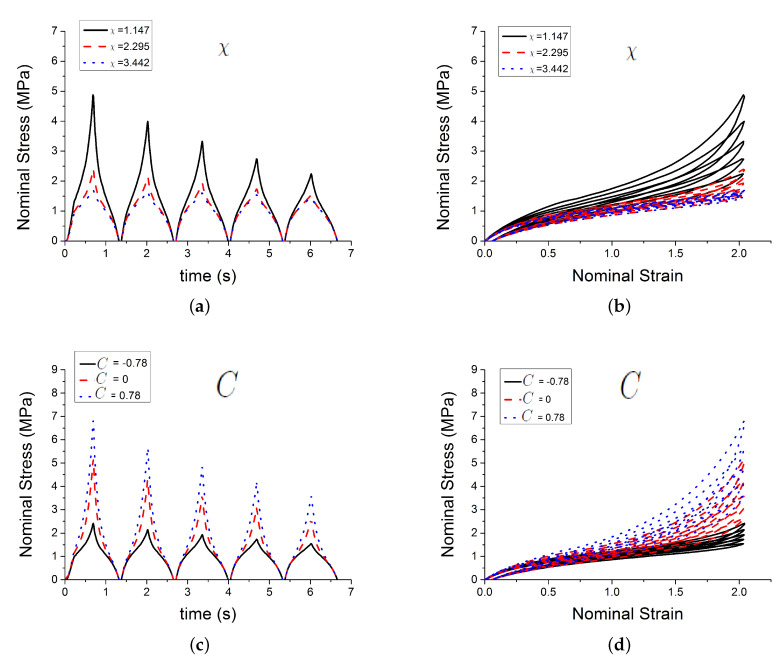

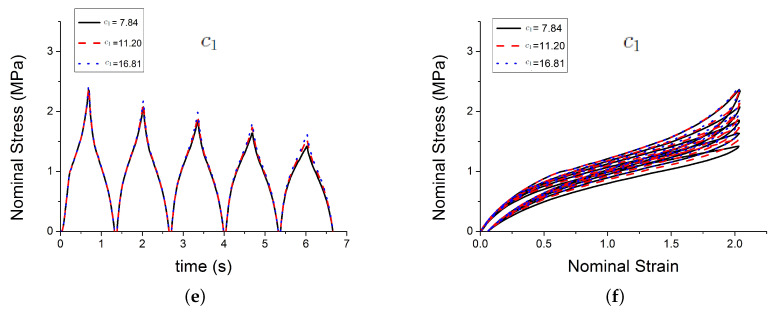
Influence of parameters. (**a**,**b**) show the effect of the parameter χ; (**c**,**d**) show the effect of the parameter *C*; and, (**e**,**f**) show the effect of the parameter $c_{1}$.

**Figure 6:**
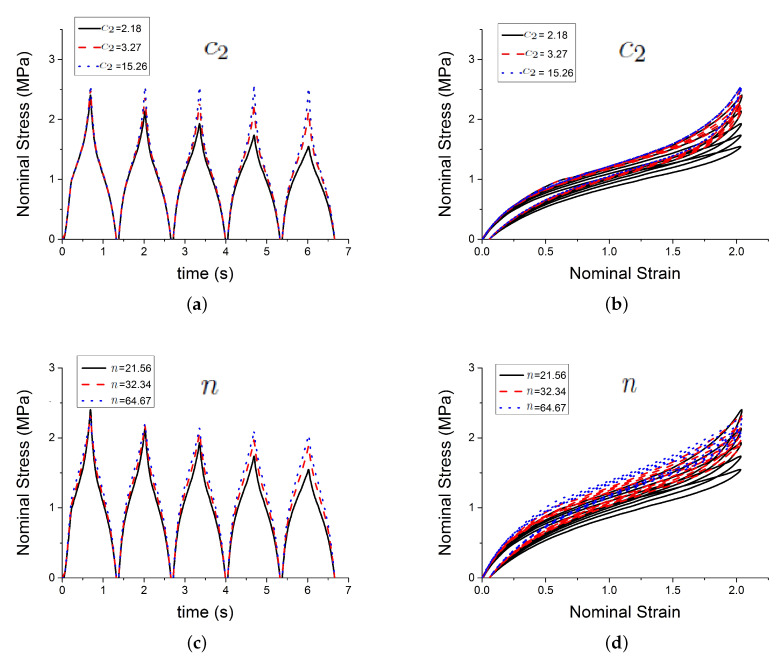

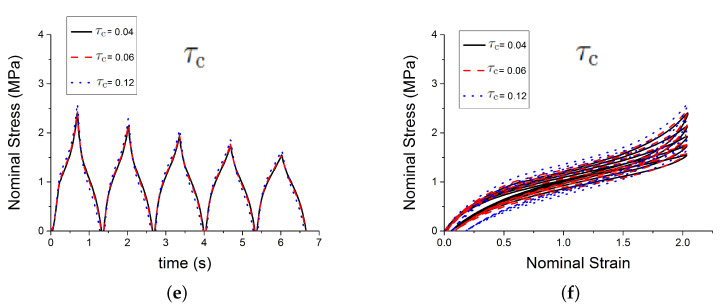
Influence of parameters. (**a**,**b**) show the effect of the parameter $c_{2}$; (**c**,**d**) show the effect of the parameter *n*; and, (**e**,**f**) show the effect of the parameter $\tau_{c}$.

**Figure 7:**
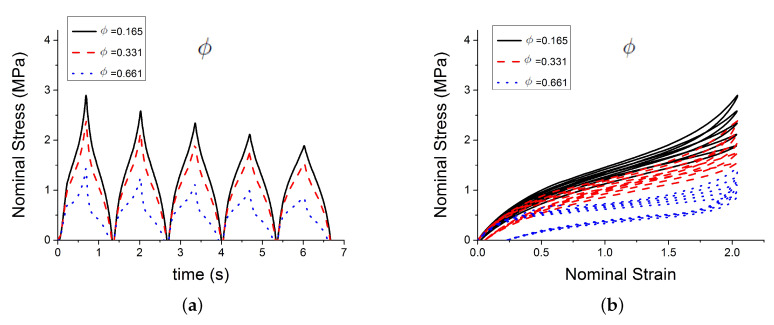
Influence of parameters. (**a**,**b**) show the effect of the parameter ϕ.

Figure 4 shows the effect of three of those parameters $G_{c}$, $G_{e}$, $X_{\max,0}$ on the predicted outputs. Figure 5 shows the effect of parameters χ, *C* and $c_{1}$. Figure 6 shows the effect of the parameters $c_{2}$, *n* and $\tau_{c}$. Figure 7 shows the effect of the parameter ϕ. $G_{c}$ and $G_{e}$, which respectively represent the crosslink modulus and entanglement modulus in the extended tube model, influence the stiffness of the material. The parameter $X_{\max,0}$ determines the value of $X_{\max}$ before any deformation in the system, and it influences cyclic stress relaxation phenomena. The parameters χ and *C* characterise the power law distribution of the amplification factor. They influence the response of the material in the loading paths, modifying the the level of stress and the dissipated energy and defining the level of continuum damage due to cyclic stress softening. *n*, $c_{1}$, and $c_{2}$ define the relaxation of the maximum amplitude factor to predict the continuum damage, so they operate on the response of the material at a strain higher than 1 and envelop the cyclic stress relaxation process. The constant $c_{2}$ scales the zero-load relaxation of the material (which is usually very small), and thus determines, together with $c_{1}$ the stress threshold to overcome before softening starts. The parameter ϕ balances elastic stresses (including softening) and viscous effects. The timescale $\tau_{c}$ defines the amount of dissipated energy.

### 4.2. Model Fit to Experimental Data

**Figure 8:**
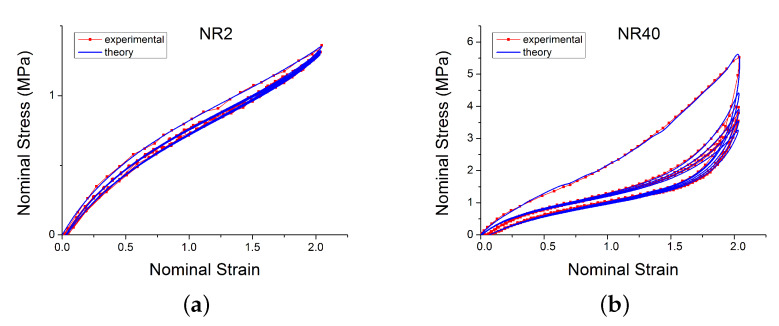

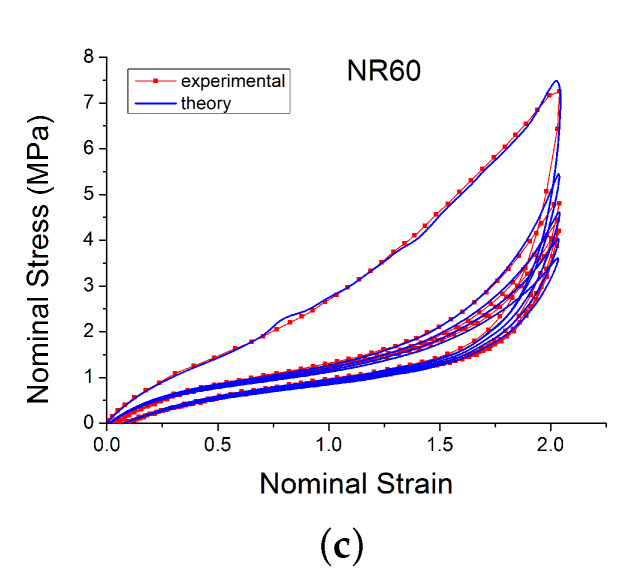
Comparison of the new model with experimental data loaded with cyclic uniaxial test with uniform maximum strain amplitude [14]. Subfigures (**a**–**c**) show respectively the behaviour for NR2, NR40 and NR60.

Model parameters were determined by minimising the mean square error between the model predictions and experimental data. Figure 8a–c show comparisons of model predictions for a selection of materials and loading configurations. For compounds with different amounts of carbon black, the model accurately reproduces the initial loading curve, relaxation cycles, the Mullins effect and the pre-strain effect.

### 4.3. Effect of Carbon Black Content on the Parameters Used in the New Model

**Figure 9:**
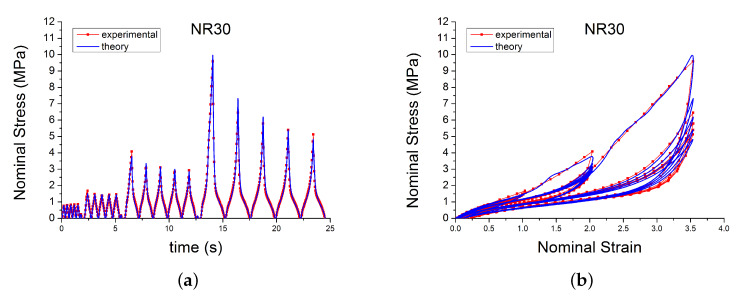
Comparison of the new model with experimental data for NR30 loaded with cyclic uniaxial stress with different maximum strain amplitude in step up. Subfigure (**a**) shows the Nominal Stress versus time. Subfigure (**b**) shows the Nominal Stress versus Nominal Strain.

The uniaxial tests, where the compound were stretched cyclically with the step up of maximum strain amplitude reached, represent a perfect test to highlight all the non linear viscoelastic behaviour under examination (Mullins effect, cyclic stress softening and permanent set) (Figure 9). The seven compounds were fitted starting from the material with the smaller amount of filler and using the new set of parameters as the guess set for the following compound. The best-fit parameters for different compounds loaded with this strain history show a robust trend with the true carbon black filler volume.

**Figure 10:**
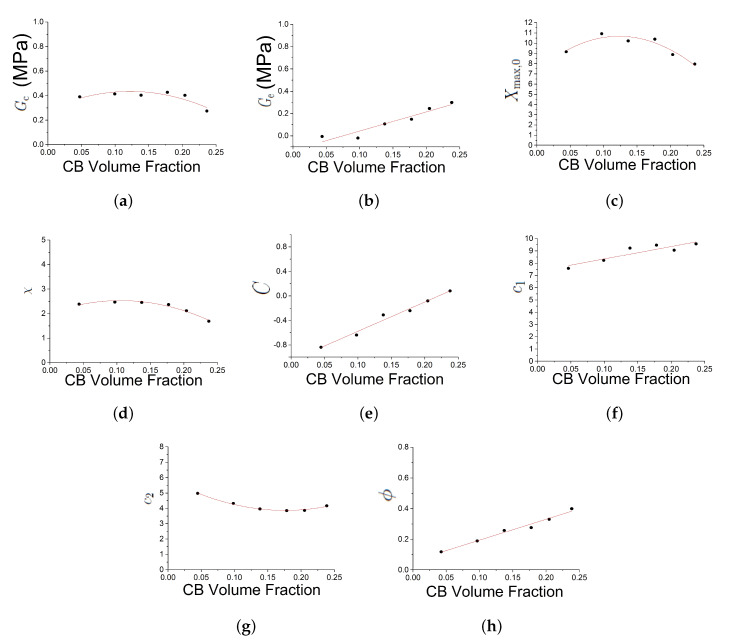

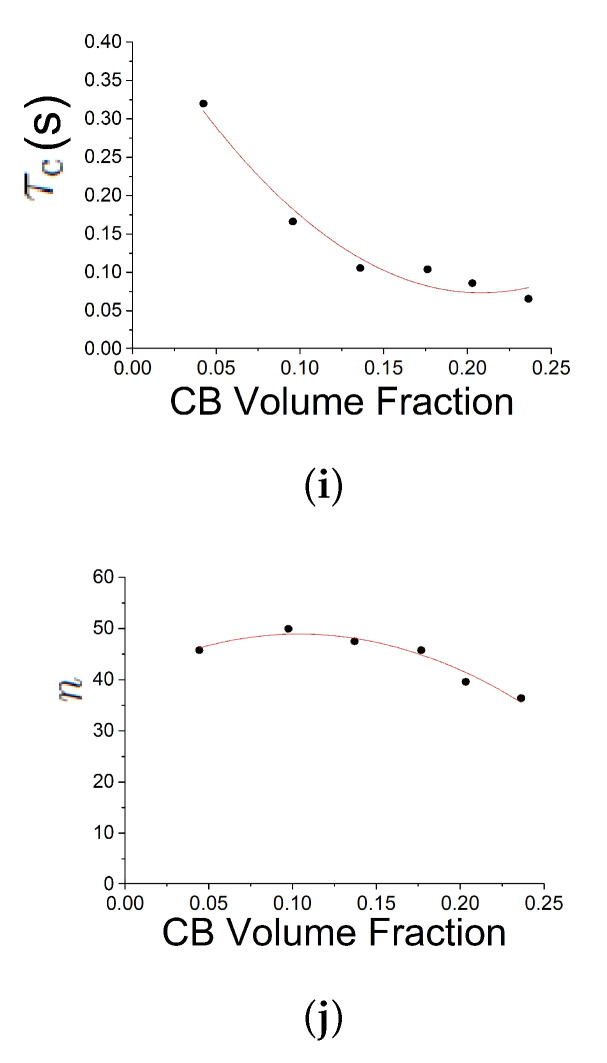
Trend of he parameters with the true carbon black filler volume $V_{f}$. Subfigures (**a**–**j**) show respectively the trend of $G_{c}$, $G_{e}$, $X_{\max,0}$, χ, *C*, $c_{1}$, $c_{2}$, ϕ, $\tau_{c}$ and *n*.

The trend of the parameters with the true Carbon Black (CB) filler volume is presented in Figure 10.

- $G_{c}$ is the modulus resulting from network crosslink and it is relatively constant with the CB volume fraction. It is found to be in a typical range for Natural Rubber vulcanizates. There is a drop in crosslink density at the highest filler loading, whose origin is not clear and may be due to experimental uncertainties and/or parameter correlations.
- $G_{e}$, the modulus resulting from network entanglement, is monotonically increasing with the CB concentration. This is surprising and lacks a clear explanation up to now. Probably this parameter somehow captures the increasing low-strain stiffness (Payne effect), even though it was not designed to fulfil this purpose.
- ϕ, represents the scaling of the elastic and inelastic part, is proportional to the true filler volume fraction.
- *n*, the distance between the network nodes is in a typical range, too. It is constant for the samples containing less than 30 vol. % of carbon black and then slightly decreases, roughly in accordance with the small drop in crosslink modulus at these filler concentrations.
- χ, the exponent of the amplification factor distribution, is approximately constant.
- $\tau_{c}$, is decreasing with filler volume fraction.
- $X_{\max,0}$ is approximately constant with filler volume fraction.
- *C* is increasing. The parameter was introduced primarily to modify the shape of the virgin loading curve. A value k>0 generates a rather linear curve, as is observed for many highly filled compounds.
- $c_{2}$ decreases with volume fraction and appears to asymptotic. From Equation (8) it can be seen that $\exp c_{2}$ defines the timescale on which $X_{\max,0}$ relaxes without load. The value obtained from fitting here create an optimal model for the timescale of the fit, but it may fail for longer simulation times. An optimal determination of $c_{2}$ and $c_{1}$ requires a stress relaxation characterisation in the fitting data.
- $c_{1}$ increases modestly with volume fraction, probably representing that the rubber-filler structures to be broken down during softening become more rigid at higher filler loadings.

Moreover, it can be seen that the parameters $G_{e}$, *C*, $c_{1}$, ϕ and $\tau_{c}$ show some kind of discontinuity in the range of 9–13 vol. % carbon black loading. This roughly corresponds to the percolation threshold of the filler network inside the polymer matrix [37].

### 4.4. Finite Element Analysis

#### Benchmark Tests

**Figure 11:**
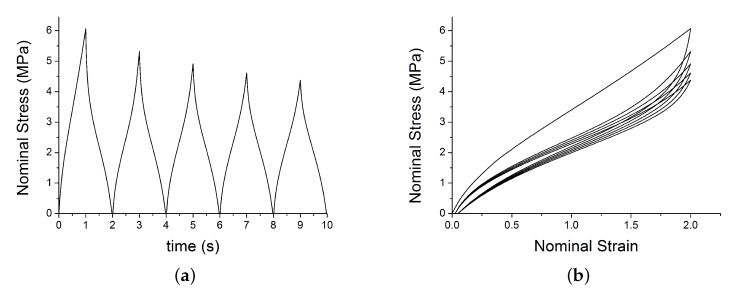
Test 1: VUMAT output: (**a**) shows the nominal stress versus time; (**b**) shows the nominal strain–stress path with the set of parameters in Table 2.

**Figure 12:**
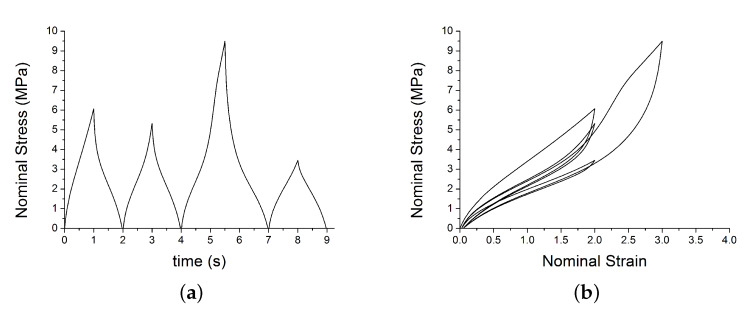
Test 2: VUMAT output: (**a**) shows the nominal stress; (**b**) shows the nominal strain–stress path with the set of parameters in Table 2.

This section shows the benchmark tests on a single element model with a simple prescribed traction loading. The model is a cube of size 0.5 mm and is meshed with 1 linear brick element with reduced integration (C3D8R). A velocity of 1 mm/s is applied in direction 1 (along the *x*-axis). The element was loaded for 5 cycles at constant max strain amplitude using 10 steps. The velocity was positive (v=1 mm/s) in the odd steps to stretch the element and negative (v=−1 mm/s) in the even steps to unload the element. The job was run using double precision. Material parameters are shown in Table 2. Figure 11 and Figure 12 show the typical output for two different cyclic loading path.

## 5. Discussion and Conclusions

**Figure 13:**
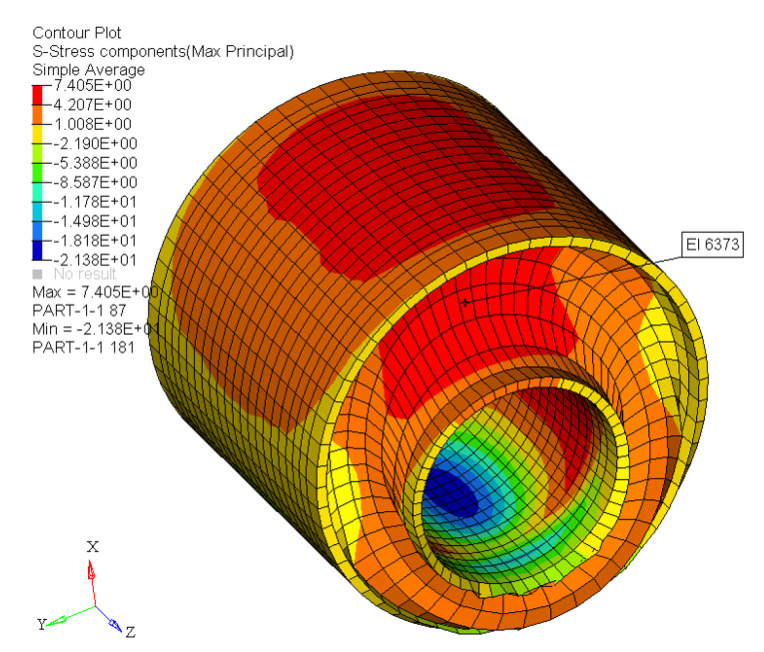
JLR toolbox: preliminary results.

The new model is based on physical rubber elasticity considerations. The total stress is the weighted sum of two contributions: an amplified, simplified extended tube model, and a hysteretic part. The hysteretic part was implemented using a single Maxwell/Prony element. For compounds with different CB volume fraction the model reproduces the non-linear behaviour under examination: Mullins effect, cyclic stress relaxation, permanent set, and hysteresis. The model requires 10 material parameters. The best-fit sets of parameters show a linear or a polynomial trend with the CB volume fraction. The sensitivity study shows the effect of altering each of the parameters. The FE implementation of the new model reproduces the expected behaviour. Finite Element Analysis was conducted on single-element models and on a realistic component. This preliminary investigation demonstrates that this model is stable when incorporated into a finite element model. The VUMAT works properly with different structures, and it is able to predict the desired non–linear viscoelastic behaviour. The new model was already implemented in the Jaguar Land Rover (JLR) suspension tool. The first job carried out a single cycle at 20 kN on a suspension loaded radially. Preliminary results are reported in Figure 13. Further studies could focus on the modeling of a broader range of deformation rates potentially requiring the use of more Prony elements. Moreover, the idea of a static hysteresis, as implemented using an intrinsic time in [28], deserves further investigation.

## Figures and Tables

**Table 1:** Compound formulation; phr (part per hundred of rubber by mass).

-	NR2	NR10	NR20	NR30	NR40	NR50	NR60
Natural Rubber, SMR CV60	100	100	100	100	100	100	100
Carbon Black, FEF N550	2	10	20	30	40	50	60
Process oil, 410	-	1	2	3	4	5	6
Zinc oxide	5	5	5	5	5	5	5
Stearic acid	2	2	2	2	2	2	2
Antioxidant/antiozonant, HPPD	3	3	3	3	3	3	3
Antiozonant wax	2	2	2	2	2	2	2
Sulfur	1.5	1.5	1.5	1.5	1.5	1.5	1.5
Accelerator, CBS	1.5	1.5	1.5	1.5	1.5	1.5	1.5
t${}_{90}$ (min)	15:16	13:50	11:50	10:30	10:00	9:04	7:10

**Table 2:** Material parameters used in the sensitivity analysis and in the VUMAT.

$G_{c}$	$G_{e}$	ϕ	$X_{\max,0}$	χ	*n*	$c_{1}$	$c_{2}$	*C*	$\tau_{c}$	*k*
0.557	0.26	0.33	11.27	2.29	21.56	11.20	2.17	−0.78	0.399	100

## Abbreviations

The following abbreviations are used in this manuscript:

DFM	Dynamic Flocculation Model
MD	Molecular Dynamics
TARRC	Tun Abdul Razac Reseach Centre
NR	Natural Rubber
SMR	Standard Malaysian Rubber
FEF	Fast Extruding Furnace
PHR	Part per Hundred Rubber
CB	Carbon Black
JLR	Jaguar Land Rover
